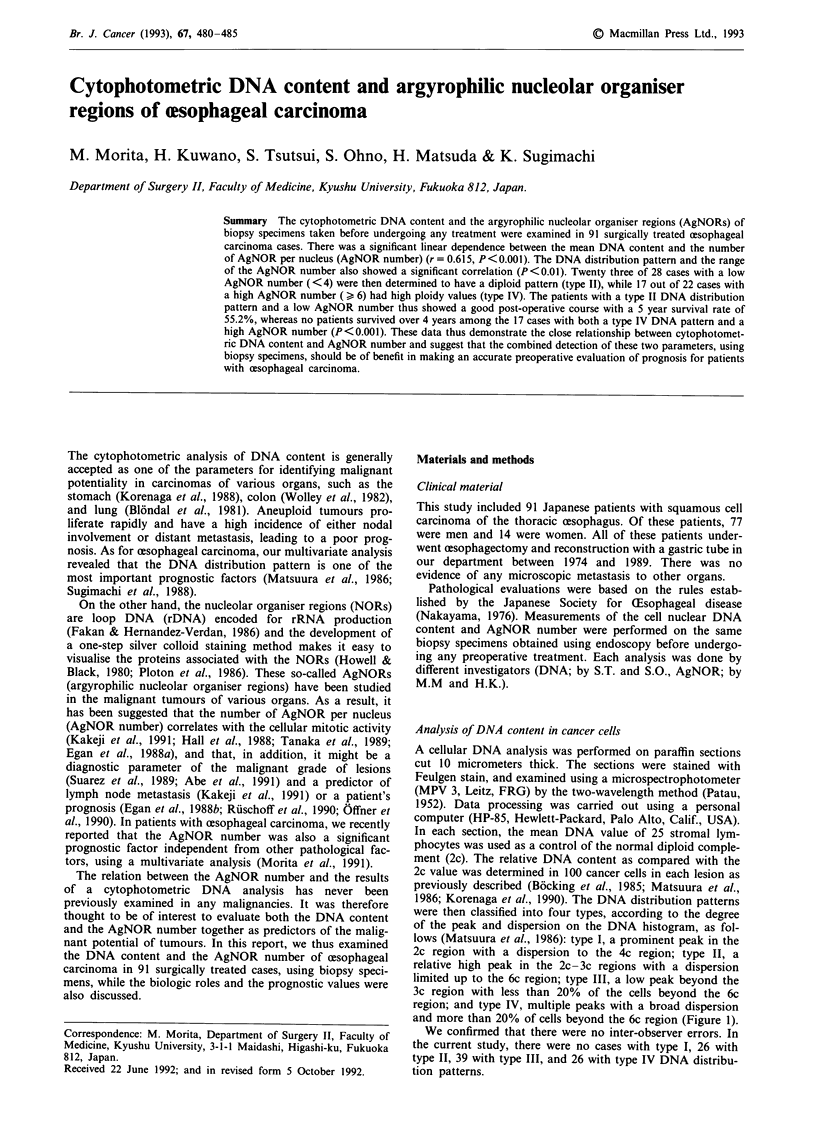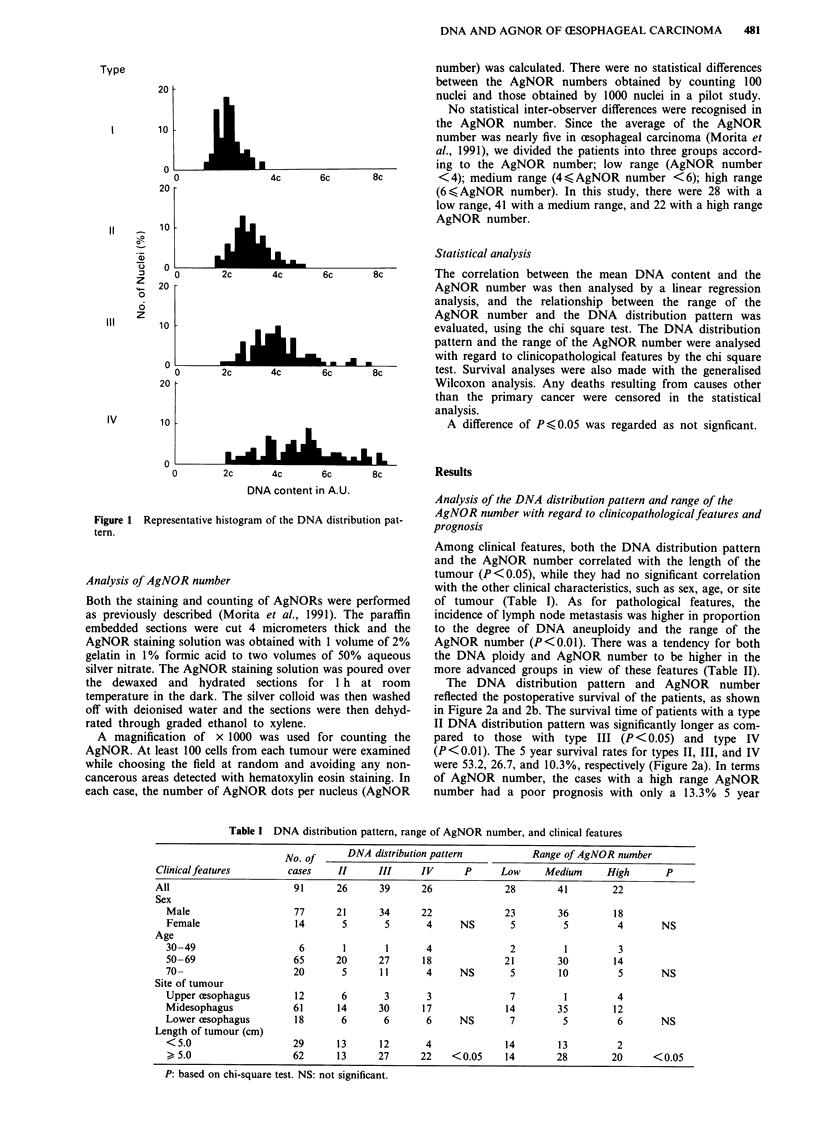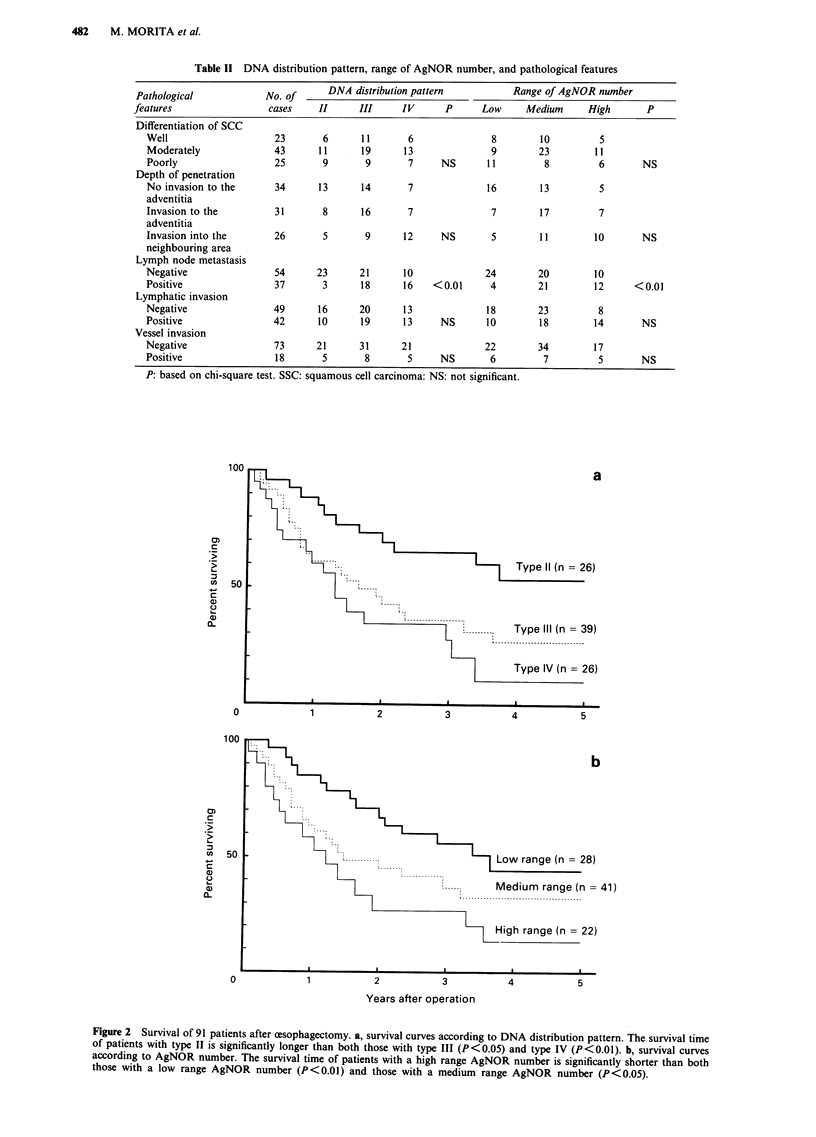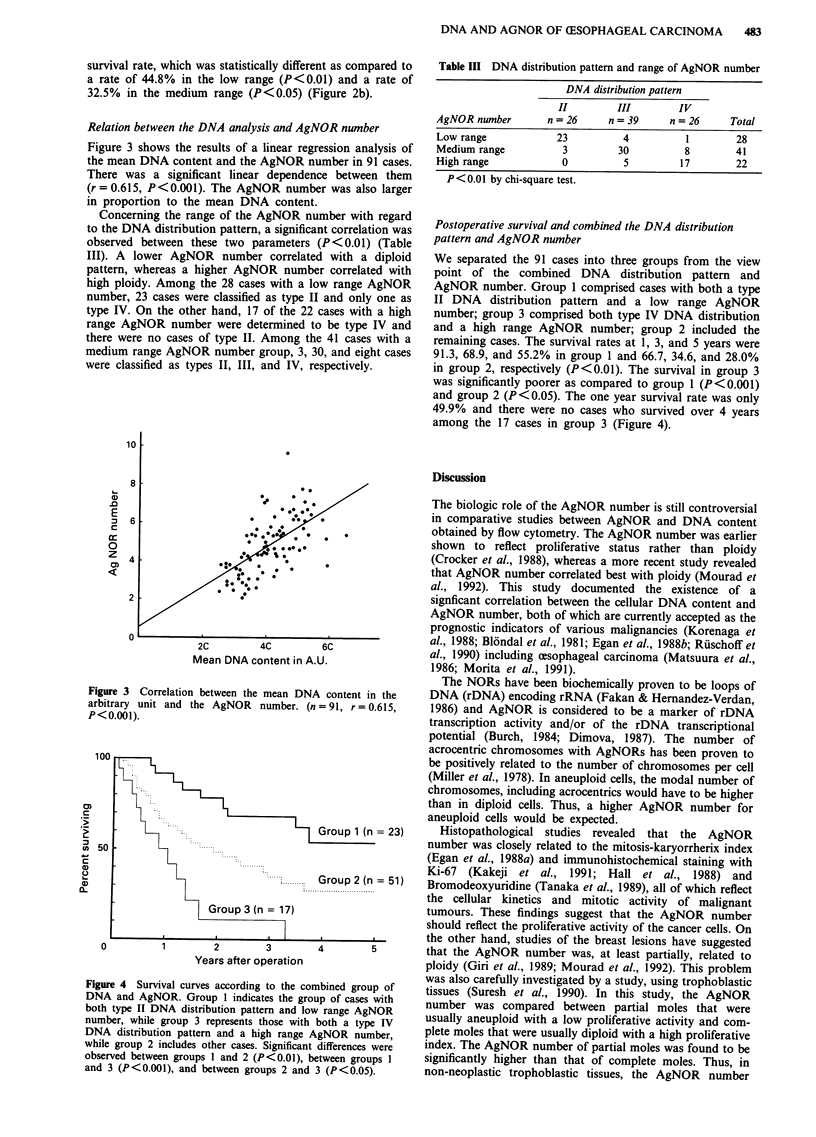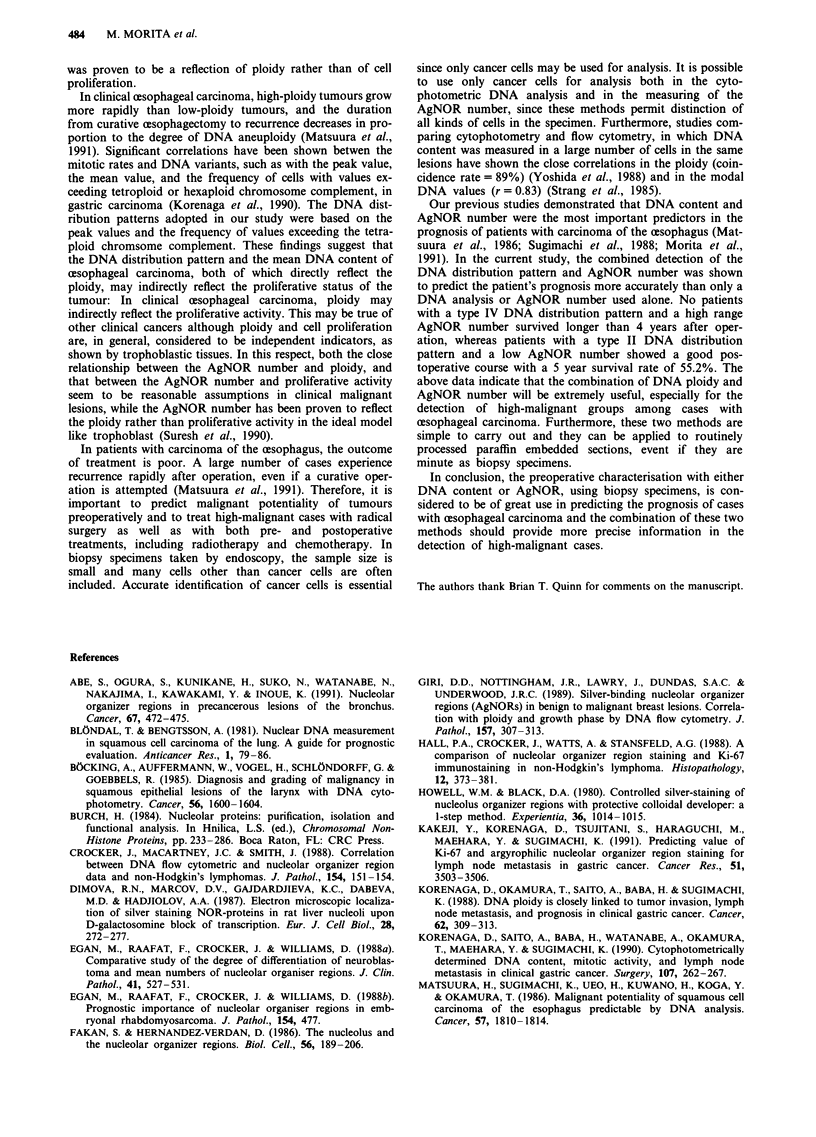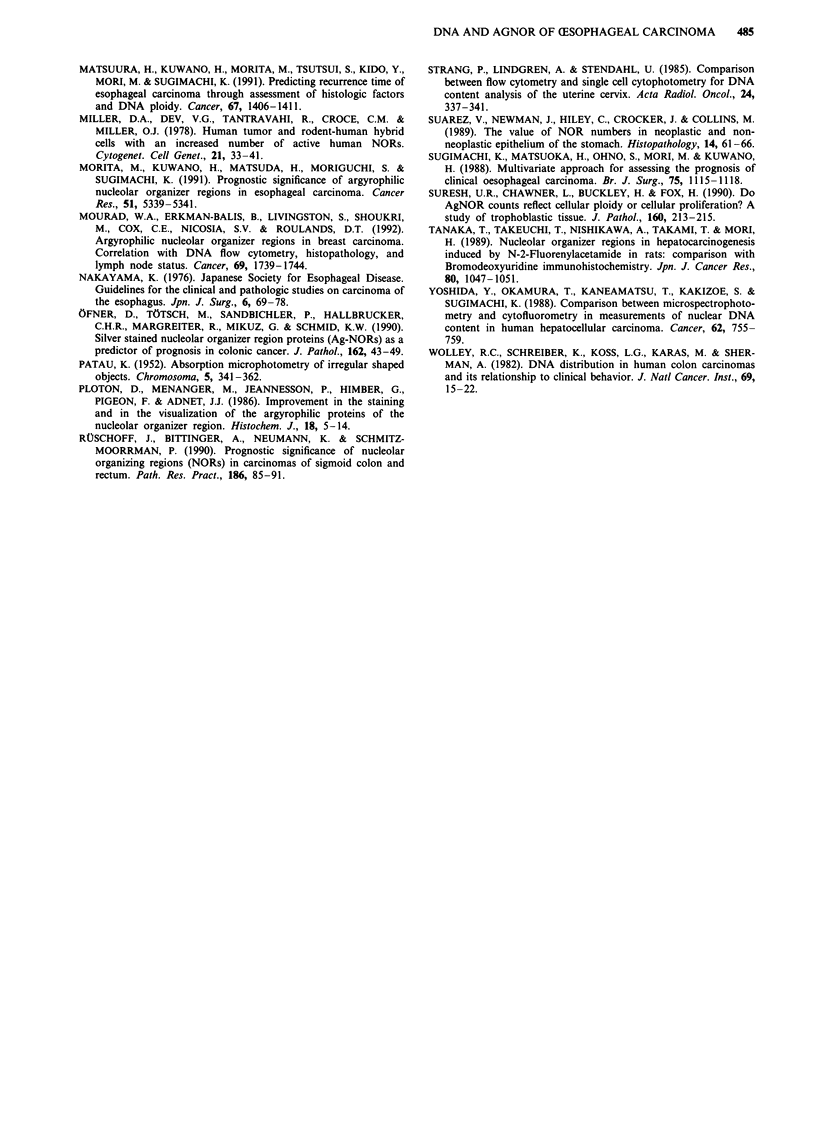# Cytophotometric DNA content and argyrophilic nucleolar organiser regions of oesophageal carcinoma.

**DOI:** 10.1038/bjc.1993.90

**Published:** 1993-03

**Authors:** M. Morita, H. Kuwano, S. Tsutsui, S. Ohno, H. Matsuda, K. Sugimachi

**Affiliations:** Department of Surgery II, Faculty of Medicine, Kyushu University, Fukuoka, Japan.

## Abstract

The cytophotometric DNA content and the argyrophilic nucleolar organiser regions (AgNORs) of biopsy specimens taken before undergoing any treatment were examined in 91 surgically treated oesophageal carcinoma cases. There was a significant linear dependence between the mean DNA content and the number of AgNOR per nucleus (AgNOR number) (r = 0.615, P < 0.001). The DNA distribution pattern and the range of the AgNOR number also showed a significant correlation (P < 0.01). Twenty three of 28 cases with a low AgNOR number (< 4) were then determined to have a diploid pattern (type II), while 17 out of 22 cases with a high AgNOR number (> or = 6) had high ploidy values (type IV). The patients with a type II DNA distribution pattern and a low AgNOR number thus showed a good post-operative course with a 5 year survival rate of 55.2%, whereas no patients survived over 4 years among the 17 cases with both a type IV DNA pattern and a high AgNOR number (P < 0.001). These data thus demonstrate the close relationship between cytophotometric DNA content and AgNOR number and suggest that the combined detection of these two parameters, using biopsy specimens, should be of benefit in making an accurate preoperative evaluation of prognosis for patients with oesophageal carcinoma.


 © Macmillan Press Ltd., 1993

### Cytophotometric DNA content and argyrophilic nucleolar organiser regions of œsophageal carcinoma

M. Morita, H. Kuwano, S. Tsutsui, S. Ohno, H. Matsuda & K. Sugimachi

*Department of Surgery II, Faculty of Medicine, Kyushu University, Fukuoka 812, Japan.*

**Summary** The cytophotometric DNA content and the argyrophilic nucleolar organiser regions (AgNORs) of biopsy specimens taken before undergoing any treatment were examined in 91 surgically treated œsophageal carcinoma cases. There was a significant linear dependence between the mean DNA content and the number of AgNOR per nucleus (AgNOR number) ($r = 0.615$, $P < 0.001$). The DNA distribution pattern and the range of the AgNOR number also showed a significant correlation ($P < 0.01$). Twenty three of 28 cases with a low AgNOR number ($< 4$) were then determined to have a diploid pattern (type II), while 17 out of 22 cases with a high AgNOR number ($\geqslant 6$) had high ploidy values (type IV). The patients with a type II DNA distribution pattern and a low AgNOR number thus showed a good post-operative course with a 5 year survival rate of 55.2%, whereas no patients survived over 4 years among the 17 cases with both a type IV DNA pattern and a high AgNOR number ($P < 0.001$). These data thus demonstrate the close relationship between cytophotometric DNA content and AgNOR number and suggest that the combined detection of these two parameters, using biopsy specimens, should be of benefit in making an accurate preoperative evaluation of prognosis for patients with œsophageal carcinoma.

The cytophotometric analysis of DNA content is generally accepted as one of the parameters for identifying malignant potentiality in carcinomas of various organs, such as the stomach (Korenaga *et al.*, 1988), colon (Wolley *et al.*, 1982), and lung (Blöndal *et al.*, 1981). Aneuploid tumours proliferate rapidly and have a high incidence of either nodal involvement or distant metastasis, leading to a poor prognosis. As for œsophageal carcinoma, our multivariate analysis revealed that the DNA distribution pattern is one of the most important prognostic factors (Matsuura *et al.*, 1986; Sugimachi *et al.*, 1988).

On the other hand, the nucleolar organiser regions (NORs) are loop DNA (rDNA) encoded for rRNA production (Fakan & Hernandez-Verdan, 1986) and the development of a one-step silver colloid staining method makes it easy to visualise the proteins associated with the NORs (Howell & Black, 1980; Ploton *et al.*, 1986). These so-called AgNORs (argyrophilic nucleolar organiser regions) have been studied in the malignant tumours of various organs. As a result, it has been suggested that the number of AgNOR per nucleus (AgNOR number) correlates with the cellular mitotic activity (Kakeji *et al.*, 1991; Hall *et al.*, 1988; Tanaka *et al.*, 1989; Egan *et al.*, 1988*a*), and that, in addition, it might be a diagnostic parameter of the malignant grade of lesions (Suarez *et al.*, 1989; Abe *et al.*, 1991) and a predictor of lymph node metastasis (Kakeji *et al.*, 1991) or a patient's prognosis (Egan *et al.*, 1988*b*; Rüschoff *et al.*, 1990; Öffner *et al.*, 1990). In patients with œsophageal carcinoma, we recently reported that the AgNOR number was also a significant prognostic factor independent from other pathological factors, using a multivariate analysis (Morita *et al.*, 1991).

The relation between the AgNOR number and the results of a cytophotometric DNA analysis has never been previously examined in any malignancies. It was therefore thought to be of interest to evaluate both the DNA content and the AgNOR number together as predictors of the malignant potential of tumours. In this report, we thus examined the DNA content and the AgNOR number of œsophageal carcinoma in 91 surgically treated cases, using biopsy specimens, while the biologic roles and the prognostic values were also discussed.

Correspondence: M. Morita, Department of Surgery II, Faculty of Medicine, Kyushu University, 3-1-1 Maidashi, Higashi-ku, Fukuoka 812, Japan.
Received 22 June 1992; and in revised form 5 October 1992.

### Materials and methods

### *Clinical material*

This study included 91 Japanese patients with squamous cell carcinoma of the thoracic œsophagus. Of these patients, 77 were men and 14 were women. All of these patients underwent œsophagectomy and reconstruction with a gastric tube in our department between 1974 and 1989. There was no evidence of any microscopic metastasis to other organs.

Pathological evaluations were based on the rules established by the Japanese Society for Œsophageal disease (Nakayama, 1976). Measurements of the cell nuclear DNA content and AgNOR number were performed on the same biopsy specimens obtained using endoscopy before undergoing any preoperative treatment. Each analysis was done by different investigators (DNA; by S.T. and S.O., AgNOR; by M.M and H.K.).

### *Analysis of DNA content in cancer cells*

A cellular DNA analysis was performed on paraffin sections cut 10 micrometers thick. The sections were stained with Feulgen stain, and examined using a microspectrophotometer (MPV 3, Leitz, FRG) by the two-wavelength method (Patau, 1952). Data processing was carried out using a personal computer (HP-85, Hewlett-Packard, Palo Alto, Calif., USA). In each section, the mean DNA value of 25 stromal lymphocytes was used as a control of the normal diploid complement (2c). The relative DNA content as compared with the 2c value was determined in 100 cancer cells in each lesion as previously described (Böcking *et al.*, 1985; Matsuura *et al.*, 1986; Korenaga *et al.*, 1990). The DNA distribution patterns were then classified into four types, according to the degree of the peak and dispersion on the DNA histogram, as follows (Matsuura *et al.*, 1986): type I, a prominent peak in the 2c region with a dispersion to the 4c region; type II, a relative high peak in the 2c–3c regions with a dispersion limited up to the 6c region; type III, a low peak beyond the 3c region with less than 20% of the cells beyond the 6c region; and type IV, multiple peaks with a broad dispersion and more than 20% of cells beyond the 6c region (Figure 1).

We confirmed that there were no inter-observer errors. In the current study, there were no cases with type I, 26 with type II, 39 with type III, and 26 with type IV DNA distribution patterns.

**Figure 1** Representative histogram of the DNA distribution pattern.

*Analysis of AgNOR number*

Both the staining and counting of AgNORs were performed as previously described (Morita *et al.*, 1991). The paraffin embedded sections were cut 4 micrometers thick and the AgNOR staining solution was obtained with 1 volume of 2% gelatin in 1% formic acid to two volumes of 50% aqueous silver nitrate. The AgNOR staining solution was poured over the dewaxed and hydrated sections for 1 h at room temperature in the dark. The silver colloid was then washed off with deionised water and the sections were then dehydrated through graded ethanol to xylene.

A magnification of × 1000 was used for counting the AgNOR. At least 100 cells from each tumour were examined while choosing the field at random and avoiding any non-cancerous areas detected with hematoxylin eosin staining. In each case, the number of AgNOR dots per nucleus (AgNOR number) was calculated. There were no statistical differences between the AgNOR numbers obtained by counting 100 nuclei and those obtained by 1000 nuclei in a pilot study.

No statistical inter-observer differences were recognised in the AgNOR number. Since the average of the AgNOR number was nearly five in œsophageal carcinoma (Morita *et al.*, 1991), we divided the patients into three groups according to the AgNOR number; low range (AgNOR number $<4$); medium range ($4 \leqslant$ AgNOR number $<6$); high range ($6 \leqslant$ AgNOR number). In this study, there were 28 with a low range, 41 with a medium range, and 22 with a high range AgNOR number.

*Statistical analysis*

The correlation between the mean DNA content and the AgNOR number was then analysed by a linear regression analysis, and the relationship between the range of the AgNOR number and the DNA distribution pattern was evaluated, using the chi square test. The DNA distribution pattern and the range of the AgNOR number were analysed with regard to clinicopathological features by the chi square test. Survival analyses were also made with the generalised Wilcoxon analysis. Any deaths resulting from causes other than the primary cancer were censored in the statistical analysis.

A difference of $P \leqslant 0.05$ was regarded as not signficant.

### Results

*Analysis of the DNA distribution pattern and range of the AgNOR number with regard to clinicopathological features and prognosis*

Among clinical features, both the DNA distribution pattern and the AgNOR number correlated with the length of the tumour ($P<0.05$), while they had no significant correlation with the other clinical characteristics, such as sex, age, or site of tumour (Table I). As for pathological features, the incidence of lymph node metastasis was higher in proportion to the degree of DNA aneuploidy and the range of the AgNOR number ($P<0.01$). There was a tendency for both the DNA ploidy and AgNOR number to be higher in the more advanced groups in view of these features (Table II).

The DNA distribution pattern and AgNOR number reflected the postoperative survival of the patients, as shown in Figure 2a and 2b. The survival time of patients with a type II DNA distribution pattern was significantly longer as compared to those with type III ($P<0.05$) and type IV ($P<0.01$). The 5 year survival rates for types II, III, and IV were 53.2, 26.7, and 10.3%, respectively (Figure 2a). In terms of AgNOR number, the cases with a high range AgNOR number had a poor prognosis with only a 13.3% 5 year

**Table I** DNA distribution pattern, range of AgNOR number, and clinical features

| *Clinical features* | *No. of cases* | *DNA distribution pattern* | | | | *Range of AgNOR number* | | | |
|---|---|---|---|---|---|---|---|---|---|
| | | *II* | *III* | *IV* | *P* | *Low* | *Medium* | *High* | *P* |
| All | 91 | 26 | 39 | 26 | | 28 | 41 | 22 | |
| Sex | | | | | | | | | |
| Male | 77 | 21 | 34 | 22 | | 23 | 36 | 18 | |
| Female | 14 | 5 | 5 | 4 | NS | 5 | 5 | 4 | NS |
| Age | | | | | | | | | |
| 30–49 | 6 | 1 | 1 | 4 | | 2 | 1 | 3 | |
| 50–69 | 65 | 20 | 27 | 18 | | 21 | 30 | 14 | |
| 70– | 20 | 5 | 11 | 4 | NS | 5 | 10 | 5 | NS |
| Site of tumour | | | | | | | | | |
| Upper œsophagus | 12 | 6 | 3 | 3 | | 7 | 1 | 4 | |
| Midesophagus | 61 | 14 | 30 | 17 | | 14 | 35 | 12 | |
| Lower œsophagus | 18 | 6 | 6 | 6 | NS | 7 | 5 | 6 | NS |
| Length of tumour (cm) | | | | | | | | | |
| $<5.0$ | 29 | 13 | 12 | 4 | | 14 | 13 | 2 | |
| $\geqslant 5.0$ | 62 | 13 | 27 | 22 | $<0.05$ | 14 | 28 | 20 | $<0.05$ |

*P*: based on chi-square test. NS: not significant.

**Table II** DNA distribution pattern, range of AgNOR number, and pathological features

| *Pathological features* | *No. of cases* | *DNA distribution pattern* II | III | IV | P | *Range of AgNOR number* Low | Medium | High | P |
|---|---|---|---|---|---|---|---|---|---|
| Differentiation of SCC | | | | | | | | | |
| Well | 23 | 6 | 11 | 6 | | 8 | 10 | 5 | |
| Moderately | 43 | 11 | 19 | 13 | | 9 | 23 | 11 | |
| Poorly | 25 | 9 | 9 | 7 | NS | 11 | 8 | 6 | NS |
| Depth of penetration | | | | | | | | | |
| No invasion to the adventitia | 34 | 13 | 14 | 7 | | 16 | 13 | 5 | |
| Invasion to the adventitia | 31 | 8 | 16 | 7 | | 7 | 17 | 7 | |
| Invasion into the neighbouring area | 26 | 5 | 9 | 12 | NS | 5 | 11 | 10 | NS |
| Lymph node metastasis | | | | | | | | | |
| Negative | 54 | 23 | 21 | 10 | | 24 | 20 | 10 | |
| Positive | 37 | 3 | 18 | 16 | <0.01 | 4 | 21 | 12 | <0.01 |
| Lymphatic invasion | | | | | | | | | |
| Negative | 49 | 16 | 20 | 13 | | 18 | 23 | 8 | |
| Positive | 42 | 10 | 19 | 13 | NS | 10 | 18 | 14 | NS |
| Vessel invasion | | | | | | | | | |
| Negative | 73 | 21 | 31 | 21 | | 22 | 34 | 17 | |
| Positive | 18 | 5 | 8 | 5 | NS | 6 | 7 | 5 | NS |

*P*: based on chi-square test. SSC: squamous cell carcinoma: NS: not significant.

**Figure 2** Survival of 91 patients after œsophagectomy. **a**, survival curves according to DNA distribution pattern. The survival time of patients with type II is significantly longer than both those with type III ($P<0.05$) and type IV ($P<0.01$). **b**, survival curves according to AgNOR number. The survival time of patients with a high range AgNOR number is significantly shorter than both those with a low range AgNOR number ($P<0.01$) and those with a medium range AgNOR number ($P<0.05$).

survival rate, which was statistically different as compared to a rate of 44.8% in the low range ($P<0.01$) and a rate of 32.5% in the medium range ($P<0.05$) (Figure 2b).

*Relation between the DNA analysis and AgNOR number*

Figure 3 shows the results of a linear regression analysis of the mean DNA content and the AgNOR number in 91 cases. There was a significant linear dependence between them ($r=0.615$, $P<0.001$). The AgNOR number was also larger in proportion to the mean DNA content.

Concerning the range of the AgNOR number with regard to the DNA distribution pattern, a significant correlation was observed between these two parameters ($P<0.01$) (Table III). A lower AgNOR number correlated with a diploid pattern, whereas a higher AgNOR number correlated with high ploidy. Among the 28 cases with a low range AgNOR number, 23 cases were classified as type II and only one as type IV. On the other hand, 17 of the 22 cases with a high range AgNOR number were determined to be type IV and there were no cases of type II. Among the 41 cases with a medium range AgNOR number group, 3, 30, and eight cases were classified as types II, III, and IV, respectively.

**Figure 3** Correlation between the mean DNA content in the arbitrary unit and the AgNOR number. ($n=91$, $r=0.615$, $P<0.001$).

**Figure 4** Survival curves according to the combined group of DNA and AgNOR. Group 1 indicates the group of cases with both type II DNA distribution pattern and low range AgNOR number, while group 3 represents those with both a type IV DNA distribution pattern and a high range AgNOR number, while group 2 includes other cases. Significant differences were observed between groups 1 and 2 ($P<0.01$), between groups 1 and 3 ($P<0.001$), and between groups 2 and 3 ($P<0.05$).

**Table III** DNA distribution pattern and range of AgNOR number

| | *DNA distribution pattern* | | | |
|---|---|---|---|---|
| *AgNOR number* | *II* *n = 26* | *III* *n = 39* | *IV* *n = 26* | *Total* |
| Low range | 23 | 4 | 1 | 28 |
| Medium range | 3 | 30 | 8 | 41 |
| High range | 0 | 5 | 17 | 22 |

$P<0.01$ by chi-square test.

*Postoperative survival and combined the DNA distribution pattern and AgNOR number*

We separated the 91 cases into three groups from the view point of the combined DNA distribution pattern and AgNOR number. Group 1 comprised cases with both a type II DNA distribution pattern and a low range AgNOR number; group 3 comprised both type IV DNA distribution and a high range AgNOR number; group 2 included the remaining cases. The survival rates at 1, 3, and 5 years were 91.3, 68.9, and 55.2% in group 1 and 66.7, 34.6, and 28.0% in group 2, respectively ($P<0.01$). The survival in group 3 was significantly poorer as compared to group 1 ($P<0.001$) and group 2 ($P<0.05$). The one year survival rate was only 49.9% and there were no cases who survived over 4 years among the 17 cases in group 3 (Figure 4).

### Discussion

The biologic role of the AgNOR number is still controversial in comparative studies between AgNOR and DNA content obtained by flow cytometry. The AgNOR number was earlier shown to reflect proliferative status rather than ploidy (Crocker *et al.*, 1988), whereas a more recent study revealed that AgNOR number correlated best with ploidy (Mourad *et al.*, 1992). This study documented the existence of a signficant correlation between the cellular DNA content and AgNOR number, both of which are currently accepted as the prognostic indicators of various malignancies (Korenaga *et al.*, 1988; Blöndal *et al.*, 1981; Egan *et al.*, 1988*b*; Rüschoff *et al.*, 1990) including œsophageal carcinoma (Matsuura *et al.*, 1986; Morita *et al.*, 1991).

The NORs have been biochemically proven to be loops of DNA (rDNA) encoding rRNA (Fakan & Hernandez-Verdan, 1986) and AgNOR is considered to be a marker of rDNA transcription activity and/or of the rDNA transcriptional potential (Burch, 1984; Dimova, 1987). The number of acrocentric chromosomes with AgNORs has been proven to be positively related to the number of chromosomes per cell (Miller *et al.*, 1978). In aneuploid cells, the modal number of chromosomes, including acrocentrics would have to be higher than in diploid cells. Thus, a higher AgNOR number for aneuploid cells would be expected.

Histopathological studies revealed that the AgNOR number was closely related to the mitosis-karyorrherix index (Egan *et al.*, 1988*a*) and immunohistochemical staining with Ki-67 (Kakeji *et al.*, 1991; Hall *et al.*, 1988) and Bromodeoxyuridine (Tanaka *et al.*, 1989), all of which reflect the cellular kinetics and mitotic activity of malignant tumours. These findings suggest that the AgNOR number should reflect the proliferative activity of the cancer cells. On the other hand, studies of the breast lesions have suggested that the AgNOR number was, at least partially, related to ploidy (Giri *et al.*, 1989; Mourad *et al.*, 1992). This problem was also carefully investigated by a study, using trophoblastic tissues (Suresh *et al.*, 1990). In this study, the AgNOR number was compared between partial moles that were usually aneuploid with a low proliferative activity and complete moles that were usually diploid with a high proliferative index. The AgNOR number of partial moles was found to be significantly higher than that of complete moles. Thus, in non-neoplastic trophoblastic tissues, the AgNOR number was proven to be a reflection of ploidy rather than of cell proliferation.

In clinical œsophageal carcinoma, high-ploidy tumours grow more rapidly than low-ploidy tumours, and the duration from curative œsophagectomy to recurrence decreases in proportion to the degree of DNA aneuploidy (Matsuura *et al.*, 1991). Significant correlations have been shown betwen the mitotic rates and DNA variants, such as with the peak value, the mean value, and the frequency of cells with values exceeding tetroploid or hexaploid chromosome complement, in gastric carcinoma (Korenaga *et al.*, 1990). The DNA distribution patterns adopted in our study were based on the peak values and the frequency of values exceeding the tetraploid chromsome complement. These findings suggest that the DNA distribution pattern and the mean DNA content of œsophageal carcinoma, both of which directly reflect the ploidy, may indirectly reflect the proliferative status of the tumour: In clinical œsophageal carcinoma, ploidy may indirectly reflect the proliferative activity. This may be true of other clinical cancers although ploidy and cell proliferation are, in general, considered to be independent indicators, as shown by trophoblastic tissues. In this respect, both the close relationship between the AgNOR number and ploidy, and that between the AgNOR number and proliferative activity seem to be reasonable assumptions in clinical malignant lesions, while the AgNOR number has been proven to reflect the ploidy rather than proliferative activity in the ideal model like trophoblast (Suresh *et al.*, 1990).

In patients with carcinoma of the œsophagus, the outcome of treatment is poor. A large number of cases experience recurrence rapidly after operation, even if a curative operation is attempted (Matsuura *et al.*, 1991). Therefore, it is important to predict malignant potentiality of tumours preoperatively and to treat high-malignant cases with radical surgery as well as with both pre- and postoperative treatments, including radiotherapy and chemotherapy. In biopsy specimens taken by endoscopy, the sample size is small and many cells other than cancer cells are often included. Accurate identification of cancer cells is essential since only cancer cells may be used for analysis. It is possible to use only cancer cells for analysis both in the cytophotometric DNA analysis and in the measuring of the AgNOR number, since these methods permit distinction of all kinds of cells in the specimen. Furthermore, studies comparing cytophotometry and flow cytometry, in which DNA content was measured in a large number of cells in the same lesions have shown the close correlations in the ploidy (coincidence rate = 89%) (Yoshida *et al.*, 1988) and in the modal DNA values ($r = 0.83$) (Strang *et al.*, 1985).

Our previous studies demonstrated that DNA content and AgNOR number were the most important predictors in the prognosis of patients with carcinoma of the œsophagus (Matsuura *et al.*, 1986; Sugimachi *et al.*, 1988; Morita *et al.*, 1991). In the current study, the combined detection of the DNA distribution pattern and AgNOR number was shown to predict the patient's prognosis more accurately than only a DNA analysis or AgNOR number used alone. No patients with a type IV DNA distribution pattern and a high range AgNOR number survived longer than 4 years after operation, whereas patients with a type II DNA distribution pattern and a low AgNOR number showed a good postoperative course with a 5 year survival rate of 55.2%. The above data indicate that the combination of DNA ploidy and AgNOR number will be extremely useful, especially for the detection of high-malignant groups among cases with œsophageal carcinoma. Furthermore, these two methods are simple to carry out and they can be applied to routinely processed paraffin embedded sections, event if they are minute as biopsy specimens.

In conclusion, the preoperative characterisation with either DNA content or AgNOR, using biopsy specimens, is considered to be of great use in predicting the prognosis of cases with œsophageal carcinoma and the combination of these two methods should provide more precise information in the detection of high-malignant cases.

The authors thank Brian T. Quinn for comments on the manuscript.

### References

ABE, S., OGURA, S., KUNIKANE, H., SUKO, N., WATANABE, N., NAKAJIMA, I., KAWAKAMI, Y. & INOUE, K. (1991). Nucleolar organizer regions in precancerous lesions of the bronchus. *Cancer*, **67**, 472–475.

BLÖNDAL, T. & BENGTSSON, A. (1981). Nuclear DNA measurement in squamous cell carcinoma of the lung. A guide for prognostic evaluation. *Anticancer Res.*, **1**, 79–86.

BÖCKING, A., AUFFERMANN, W., VOGEL, H., SCHLÖNDORFF, G. & GOEBBELS, R. (1985). Diagnosis and grading of malignancy in squamous epithelial lesions of the larynx with DNA cytophotometry. *Cancer*, **56**, 1600–1604.

BURCH, H. (1984). Nucleolar proteins: purification, isolation and functional analysis. In Hnilica, L.S. (ed.), *Chromosomal Non-Histone Proteins*, pp. 233–286. Boca Raton, FL: CRC Press.

CROCKER, J., MACARTNEY, J.C. & SMITH, J. (1988). Correlation between DNA flow cytometric and nucleolar organizer region data and non-Hodgkin's lymphomas. *J. Pathol.*, **154**, 151–154.

DIMOVA, R.N., MARCOV, D.V., GAJDARDJIEVA, K.C., DABEVA, M.D. & HADJIOLOV, A.A. (1987). Electron microscopic localization of silver staining NOR-proteins in rat liver nucleoli upon D-galactosomine block of transcription. *Eur. J. Cell Biol.*, **28**, 272–277.

EGAN, M., RAAFAT, F., CROCKER, J. & WILLIAMS, D. (1988*a*). Comparative study of the degree of differentiation of neuroblastoma and mean numbers of nucleolar organiser regions. *J. Clin. Pathol.*, **41**, 527–531.

EGAN, M., RAAFAT, F., CROCKER, J. & WILLIAMS, D. (1988*b*). Prognostic importance of nucleolar organiser regions in embryonal rhabdomyosarcoma. *J. Pathol.*, **154**, 477.

FAKAN, S. & HERNANDEZ-VERDAN, D. (1986). The nucleolus and the nucleolar organizer regions. *Biol. Cell.*, **56**, 189–206.

GIRI, D.D., NOTTINGHAM, J.R., LAWRY, J., DUNDAS, S.A.C. & UNDERWOOD, J.R.C. (1989). Silver-binding nucleolar organizer regions (AgNORs) in benign to malignant breast lesions. Correlation with ploidy and growth phase by DNA flow cytometry. *J. Pathol.*, **157**, 307–313.

HALL, P.A., CROCKER, J., WATTS, A. & STANSFELD, A.G. (1988). A comparison of nucleolar organizer region staining and Ki-67 immunostaining in non-Hodgkin's lymphoma. *Histopathology*, **12**, 373–381.

HOWELL, W.M. & BLACK, D.A. (1980). Controlled silver-staining of nucleolus organizer regions with protective colloidal developer: a 1-step method. *Experientia*, **36**, 1014–1015.

KAKEJI, Y., KORENAGA, D., TSUJITANI, S., HARAGUCHI, M., MAEHARA, Y. & SUGIMACHI, K. (1991). Predicting value of Ki-67 and argyrophilic nucleolar organizer region staining for lymph node metastasis in gastric cancer. *Cancer Res.*, **51**, 3503–3506.

KORENAGA, D., OKAMURA, T., SAITO, A., BABA, H. & SUGIMACHI, K. (1988). DNA ploidy is closely linked to tumor invasion, lymph node metastasis, and prognosis in clinical gastric cancer. *Cancer*, **62**, 309–313.

KORENAGA, D., SAITO, A., BABA, H., WATANABE, A., OKAMURA, T., MAEHARA, Y. & SUGIMACHI, K. (1990). Cytophotometrically determined DNA content, mitotic activity, and lymph node metastasis in clinical gastric cancer. *Surgery*, **107**, 262–267.

MATSUURA, H., SUGIMACHI, K., UEO, H., KUWANO, H., KOGA, Y. & OKAMURA, T. (1986). Malignant potentiality of squamous cell carcinoma of the esophagus predictable by DNA analysis. *Cancer*, **57**, 1810–1814.

MATSUURA, H., KUWANO, H., MORITA, M., TSUTSUI, S., KIDO, Y., MORI, M. & SUGIMACHI, K. (1991). Predicting recurrence time of esophageal carcinoma through assessment of histologic factors and DNA ploidy. *Cancer*, **67**, 1406–1411.

MILLER, D.A., DEV, V.G., TANTRAVAHI, R., CROCE, C.M. & MILLER, O.J. (1978). Human tumor and rodent-human hybrid cells with an increased number of active human NORs. *Cytogenet. Cell Genet.*, **21**, 33–41.

MORITA, M., KUWANO, H., MATSUDA, H., MORIGUCHI, S. & SUGIMACHI, K. (1991). Prognostic significance of argyrophilic nucleolar organizer regions in esophageal carcinoma. *Cancer Res.*, **51**, 5339–5341.

MOURAD, W.A., ERKMAN-BALIS, B., LIVINGSTON, S., SHOUKRI, M., COX, C.E., NICOSIA, S.V. & ROULANDS, D.T. (1992). Argyrophilic nucleolar organizer regions in breast carcinoma. Correlation with DNA flow cytometry, histopathology, and lymph node status. *Cancer*, **69**, 1739–1744.

NAKAYAMA, K. (1976). Japanese Society for Esophageal Disease. Guidelines for the clinical and pathologic studies on carcinoma of the esophagus. *Jpn. J. Surg.*, **6**, 69–78.

ÖFNER, D., TÖTSCH, M., SANDBICHLER, P., HALLBRUCKER, C.H.R., MARGREITER, R., MIKUZ, G. & SCHMID, K.W. (1990). Silver stained nucleolar organizer region proteins (Ag-NORs) as a predictor of prognosis in colonic cancer. *J. Pathol.*, **162**, 43–49.

PATAU, K. (1952). Absorption microphotometry of irregular shaped objects. *Chromosoma*, **5**, 341–362.

PLOTON, D., MENANGER, M., JEANNESSON, P., HIMBER, G., PIGEON, F. & ADNET, J.J. (1986). Improvement in the staining and in the visualization of the argyrophilic proteins of the nucleolar organizer region. *Histochem. J.*, **18**, 5–14.

RÜSCHOFF, J., BITTINGER, A., NEUMANN, K. & SCHMITZ-MOORRMAN, P. (1990). Prognostic significance of nucleolar organizing regions (NORs) in carcinomas of sigmoid colon and rectum. *Path. Res. Pract.*, **186**, 85–91.

STRANG, P., LINDGREN, A. & STENDAHL, U. (1985). Comparison between flow cytometry and single cell cytophotometry for DNA content analysis of the uterine cervix. *Acta Radiol. Oncol.*, **24**, 337–341.

SUAREZ, V., NEWMAN, J., HILEY, C., CROCKER, J. & COLLINS, M. (1989). The value of NOR numbers in neoplastic and non-neoplastic epithelium of the stomach. *Histopathology*, **14**, 61–66.

SUGIMACHI, K., MATSUOKA, H., OHNO, S., MORI, M. & KUWANO, H. (1988). Multivariate approach for assessing the prognosis of clinical oesophageal carcinoma. *Br. J. Surg.*, **75**, 1115–1118.

SURESH, U.R., CHAWNER, L., BUCKLEY, H. & FOX, H. (1990). Do AgNOR counts reflect cellular ploidy or cellular proliferation? A study of trophoblastic tissue. *J. Pathol.*, **160**, 213–215.

TANAKA, T., TAKEUCHI, T., NISHIKAWA, A., TAKAMI, T. & MORI, H. (1989). Nucleolar organizer regions in hepatocarcinogenesis induced by N-2-Fluorenylacetamide in rats: comparison with Bromodeoxyuridine immunohistochemistry. *Jpn. J. Cancer Res.*, **80**, 1047–1051.

YOSHIDA, Y., OKAMURA, T., KANEAMATSU, T., KAKIZOE, S. & SUGIMACHI, K. (1988). Comparison between microspectrophotometry and cytofluorometry in measurements of nuclear DNA content in human hepatocellular carcinoma. *Cancer*, **62**, 755–759.

WOLLEY, R.C., SCHREIBER, K., KOSS, L.G., KARAS, M. & SHERMAN, A. (1982). DNA distribution in human colon carcinomas and its relationship to clinical behavior. *J. Natl Cancer. Inst.*, **69**, 15–22.